# Interstitial Extra-Uterine Gestation

**Published:** 1848-06

**Authors:** 


*Interstitial Extra-Uterine Gestation.*—The following case is related by Dr. Payan, of Aix. It was that of a woman, aged thirty-two. Her pregnancy was of three months' duration, and she enjoyed good health. Suddenly, however, and without any appreciable cause, she became ill, was seized with violent pains in the hypogastric region, burning thirst, and extreme prostration; soon syncope occurred, and she died from this about nine hours after the time when the illness first commenced. On the post-mortem examination, a large quantity of coagulated blood was found covering the uterus. This organ was of larger size than in the unimpregnated state, and presented a prominence at its upper part, in part diaphanous, and through which an embryo could be perceived. On opening the uterus, its cavity was found to be large, and lined throughout with a kind of false membrane, incompletely organized, not containing any fœtus, and also devoid of any blood. It evidently represented the decidua vera. Projecting above, from the uterine cavity at the fundus uteri, on the left side, was another cavity, in the neighborhood of the uterine termination of the Fallopian tube, and probably communicating with it. This second cavity was formed out of the thickness, and at the expense, of the walls of the fundus; its own parietes were thin, and, in parts, translucid. It was in this secondary and interstitial pouch that the entire fœtus, with its placenta, was lodged.

This case was therefore one of extra-uterine gestation of an uncommon kind. Development had proceeded till the third month, when the embryo produced some rupture which led to fatal hæmorrhage. Two other forms of extra-uterine development are known—one in the Fallopian tube itself, the other in the ovary; but in the former, a fatal issue sooner supervenes than in the ovarian or in the interstitial variety. When rupture of the enclosing fœtal sac takes place, the fœtus will in most cases escape into the abdominal cavity, but in the instance narrated it appears to have retained its position in its unnatural cavity. Some describe a form of extra-uterine fœtation, where the fœtus is developed in the abdominal cavity, supposing the ovum to escape the grasp of the fimbriated extremity of the Fallopian tube, in its passage into it from the ovary, and to fall into that cavity.

Such cases as the above are beyond the remedial power of art, and generally prove rapidly fatal by hæmorrhage and serous inflammation.

Looked upon in a medico-legal point of view, the only suggestion that can be made contrary to the generally received opinion of the mode and cause of death is, that an instrument has been passed between the uterus and fœtal membranes, suffering the entire ovum to escape, and inducing contraction of the uterus, and rupture. But the appearances presented on a post-mortem examination will at any time prove or disprove the truth of any such assumption.—*London Lancet.*

